# Preface to *Journal of Dermatology* special issue: Neutrophilic dermatoses and autoinflammation

**DOI:** 10.1111/1346-8138.17104

**Published:** 2024-01-09

**Authors:** Toshiyuki Yamamoto

**Affiliations:** ^1^ Department of Dermatology Fukushima Medical University Fukushima Japan

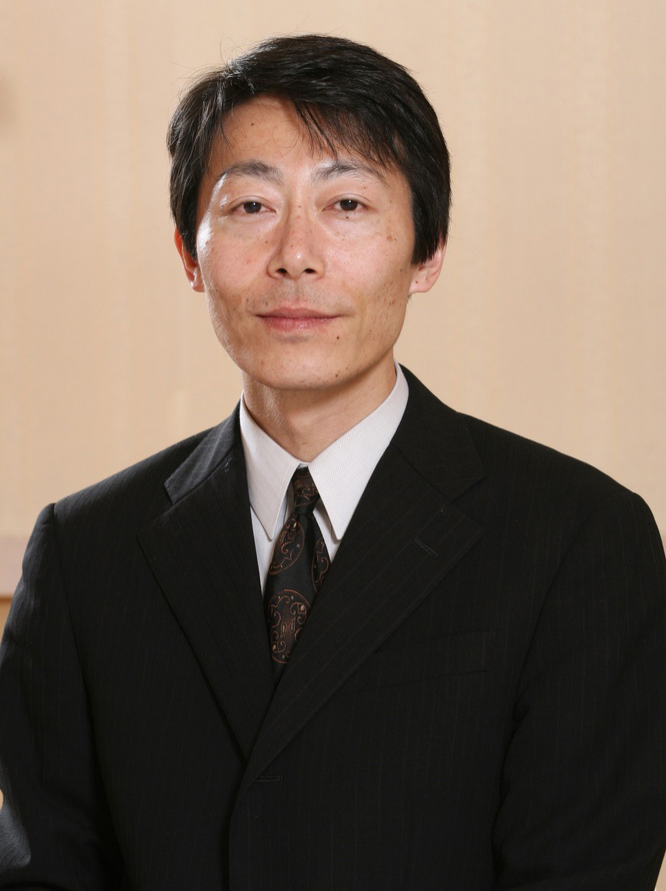

Autoinflammatory syndrome is caused by hyperactivation of the innate immune system, characterized by recurrent episodes of fever with systemic/organ‐specific inflammation. Uncontrolled activation of the innate immune response drives inflammatory cytokine pathways in the pathophysiology of autoinflammatory syndromes/diseases. Since various skin symptoms/diseases are occasionally included in autoinflammatory syndromes, dermatologists may be in an advantageous position to detect, or at least suspect, autoinflammatory syndromes. In this review, current knowledge on skin manifestation, pathogenesis, and therapies for several autoinflammatory diseases are discussed from the dermatological viewpoint.

Chen focuses on cutaneous vasculitis in familial Mediterranean fever, deficiency of adenosine deaminase type 2 and other topics such as Sweet syndrome and VEXAS syndrome. It is known that Sweet syndrome and relapsing polychondritis are occasionally associated in a single patient, and the etiology is believed to be shared by activated neutrophils. It is easy to assume that many such cases can now be diagnosed as VEXAS syndrome. Neutrophilic dermatoses include several disorders, and pyoderma gangrenosum and Sweet syndrome are the representative diseases. Activated neutrophils recruit not only into the skin but also other organs and induce organ inflammation or aseptic abscess. There are several pyogenic arthritis, pyoderma gangrenosum and acne (PAPA) spectrum syndromes. Pyoderma gangrenosum and acne are commonly observed in PAPA syndrome, pyoderma gangrenosum, acne, and suppurative hidradenitis (PASH) syndrome, and pyogenic arthritis, pyoderma gangrenosum, acne, hidradenitis suppurativa **(**PAPASH) syndrome. Satoh discusses current knowledge on identified mutations and the presumed pathogenesis of neutrophilic dermatoses. In pyoderma gangrenosum and hidradenitis suppurativa, inflammatory cytokines such as tumor necrosis factor‐α, interleukin (IL)‐1, IL‐8, IL‐17, and iIL‐23. Yamanaka reviews the current therapeutic approach to neutrophilic dermatosis, that target those specific cytokines. Finally, systemic and cutaneous lupus are highlighted. Neutrophils, dendritic cells, T cells, and B cells are important in lupus pathogenesis. Neutrophil infiltration was observed in several types of cutaneous lupus erythematosus, and neutrophil extracellular traps are important, not only in host defense but also inflammation, innate immunity, autoimmunity, allergy, and vasculitis/vasculopathy.

Other than the above‐mentioned disorders, although rare, Blau syndrome and Aicardi‐Goutières syndrome exhibit unique skin manifestations, through which those syndromes can be suspected. Recent understanding suggests that SAPHO syndrome is included as an autoinflammatory syndrome. By contrast, pustulotic arthro‐osteitis in association with palmoplantar pustulosis is conventionally called SAPHO syndrome. Although pustulotic arthro‐osteitis is frequently observed in Japan, I believe that true SAPHO syndrome is extremely rare. Autoinflammatory diseases/syndromes are increasing in number. I hope that the readers of the *Journal of Dermatology* will be much interested in the autoimmune aspects of several disorders with excessive innate immune response, other than inherited autoinflammatory syndromes.

